# Spontaneous rupture of the ovarian vein in association with nutcracker syndrome: a case report

**DOI:** 10.1186/s13256-021-03192-8

**Published:** 2021-12-18

**Authors:** Akihito Yamamoto, Seiryu Kamoi, Shunji Suzuki

**Affiliations:** Department of Obstetrics and Gynecology, Nippon Medical School, 1-1-5 Sendagi, Bunkyo-ku, Tokyo, 113-8602 Japan

**Keywords:** Nutcracker syndrome, Nutcracker phenomenon, Pelvic congestion syndrome, Ovarian vein, Gonadal vein, Pelvic venous plexus, Rupture, Endovascular treatment, Vascular embolization, Diagnostic imaging

## Abstract

**Background:**

Nutcracker syndrome is a condition in which the left renal vein is pinched between the abdominal aorta and the superior mesenteric artery, resulting in an increase in renal vein pressure and certain symptoms. We report a very rare case of retroperitoneal hematoma caused by the rupture of varicose veins of the left ovary.

**Case presentation:**

A 77-year-old Japanese woman, para 7, experienced sudden left lower abdominal pain. She had no history of trauma or treatment complications. Computed tomography revealed a left retroperitoneal hematoma, but her abdominal pain subsided quickly; thus, urgent treatment was not required. We then scheduled her for an assessment regarding the cause of her bleeding. However, 6 days after the pain onset, abdominal pain symptoms recurred, confirming hematoma regrowth. Magnetic resonance imaging and three-dimensional computed tomography revealed an abnormal vascular network from the left side of the uterus to the left adnexa. Subsequent angiography revealed that the retroperitoneal bleeding originated from rupture of the distended left ovarian vein, which caused blood reflux from the left renal vein to the left ovarian vein. Although angiography confirmed a passage between the left renal vein and inferior vena cava, computed tomography showed obvious stenosis in the left renal vein. In accordance with these findings, we diagnosed the cause of the distention and rupture of the left ovarian vein as nutcracker syndrome. She underwent embolization of the left ovarian vein as hemostasis treatment, and had a good course thereafter.

**Conclusions:**

This is the first report of a spontaneous rupture of the left ovarian vein caused by nutcracker syndrome. Nutcracker syndrome is not yet well known to clinicians and should be considered as part of the differential diagnosis when an abnormal vascular network in the pelvis is found.

## Background

The left renal vein (LRV) anatomically passes between the abdominal aorta and the superior mesenteric artery (SMA). The nutcracker phenomenon is the condition in which this gap narrows for some reason and causes stenosis in the LRV, and when it is accompanied by certain clinical symptoms, it is called nutcracker syndrome (NCS) [1, 2].

In 1937, the anatomist John C. Boileau Grant first reported the phenomenon of LRV pinching, and in 1972, de Schepper named the condition “nutcracker syndrome” [1, 3–5]. Because of its short history, NCS has not been thoroughly investigated and is not recognized by clinicians.

Elevated blood pressure in the LRV causes certain signs and symptoms of NCS, hematuria being the most common, but distension and dysfunction of the gonadal vein also cause pelvic congestion syndrome (PCS) in women and varicocele in men. In recent years, there have some reports on NCS but, to our knowledge, this report is the first that focuses on spontaneous rupture of the ovarian vein caused by NCS.

We report this case to raise awareness of the pathophysiology of NCS, and to suggest that ovarian varicose veins may rupture spontaneously.

## Case presentation

A 77-year-old Japanese woman, para 7, who went through menopause at age 48, suffered sudden onset of left lower abdominal pain and visited a primary care doctor. Computed tomography (CT) revealed torsion of a left ovarian cyst, and she was transferred to our hospital for surgery. She had a history of left-side breast cancer and underwent total left mastectomy at the age of 67, with no recurrence thereafter. Although she was a carrier of hepatitis C, her liver function and coagulation remained normal, and she was being followed-up without medication. Furthermore, she was taking nifedipine and candesartan cilexetil for hypertension. Meanwhile, her family history and psychosocial history were unremarkable. She also had no history of trauma.

**Fig. 1:**
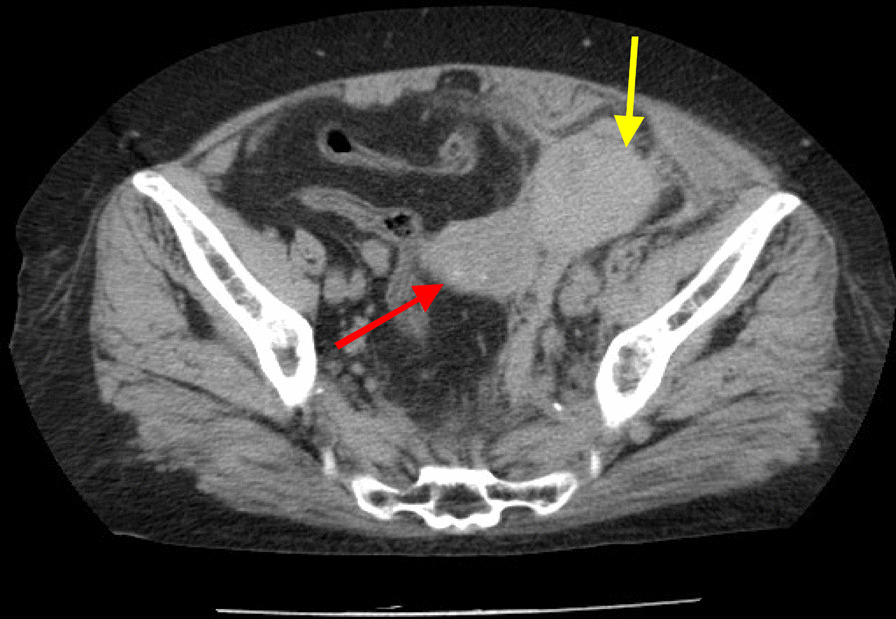
Computed tomographic image at the time of the first hospitalization. The red arrow indicates the uterus and the yellow arrow indicates the left pelvic retroperitoneal hematoma. Initially, this hematoma was diagnosed as a left ovarian cyst

**Fig. 2:**
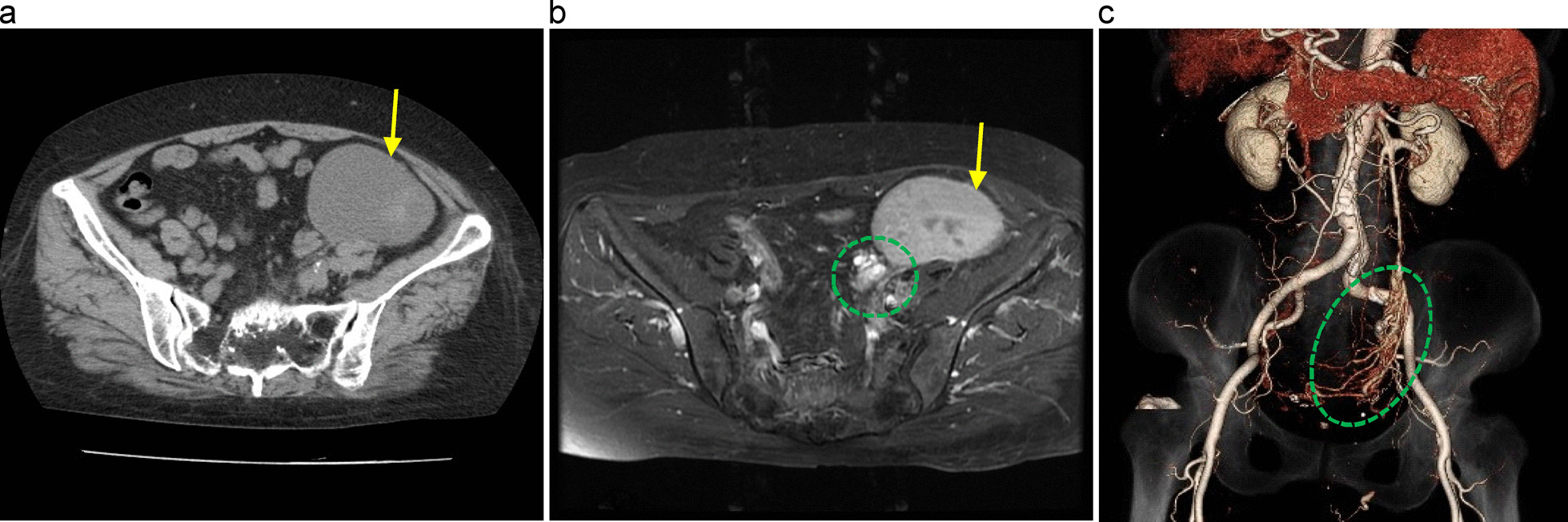
Images obtained at the time of readmission. **a** Computed tomography (CT) showed the 11.7 cm hematoma and a faint area of high absorption inside that was suggestive of rebleeding. **b** Magnetic resonance imaging; arteriovenous malformation or fistula was suspected because of the accumulation of vascular structures between the uterus and the mass. **c** Three-dimensional CT depicted an abnormal vascular network from the left side of the uterus to the left adnexa. A short-circuit inflow from the uterine artery to the ovarian vein was suspected. All yellow arrows indicate the hematoma and dotted green lines indicate the abnormal vascular network

CT showed a 7.5-cm long elliptical mass in the left adnexal region that was continuous with the uterus (Fig. 1). The ovary was atrophic because of the patient’s age, and difficult to identify; the appearance of blood and the continuity with the surrounding pelvic peritoneum were suggestive of retroperitoneal hematoma. Physical findings at admission were as follows: height, 144.5 cm; weight, 57.2 kg; body mass index (BMI), 27.4 kg/m^2^; blood pressure, 112/50 mm Hg; pulse, 72 bpm; and body temperature, 37.1 °C. Physical examination detected no significant findings. On external (body surface) and internal examinations, no palpable masses or tenderness were noted in the left pelvic area. Transvaginal ultrasound revealed an atrophied uterus; however, the bilateral adnexa could not be identified due to atrophy. The hematoma in the left pelvis could be identified. These findings were identical to those obtained by CT. Blood test results were a hemoglobin value of 9.5 g/dL, a hematocrit value of 28.4%, a white blood cell count of 7970/μL, and a C-reactive protein level of 0.05 mg/dL. Urinalysis revealed mild occult blood. After admission, the hematoma did not enlarge, and the hemoglobin and hematocrit levels did not decrease. After 11 days of bed rest, with no exacerbation of the inflammatory response, lower abdominal pain was relieved, and she was discharged. After discharge, we planned to investigate the cause of her bleeding on an outpatient basis, but 6 days after discharge, she was readmitted to our hospital after relapse of symptoms caused by retroperitoneal bleeding from the same site. The hematoma had grown to a size of 11.7 cm, and magnetic resonance imaging showed an abnormal vascular network extending from the left side of the uterus to the left adnexal area. Three-dimensional CT (3D-CT) similarly showed an abnormal vascular network on the left side of the uterus, and we suspected a possible arteriovenous malformation (AVM) or arteriovenous fistula (AVF) from the uterine artery to the left ovarian vein (LOV; Fig. 2).

**Fig. 3:**
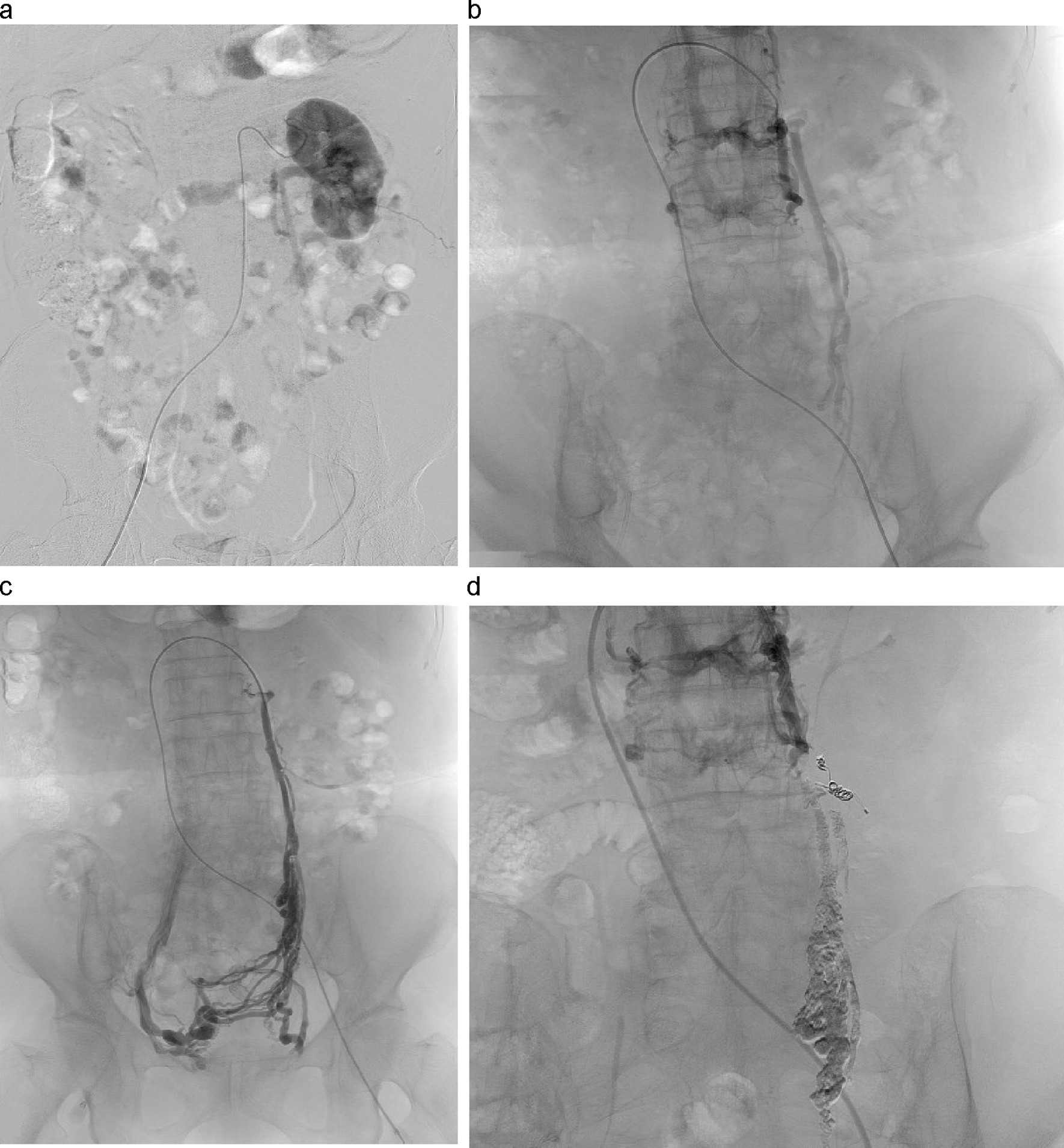
Angiographic images. **a** Left renal arteriography showed that venous blood did not flow into the inferior vena cava but flowed back into the ovarian vein. **b** Left renal venography showed blood flow to the left ovarian vein and lumbar vein. **c** Left ovarian venography showed that reflux blood flowed into the right internal iliac vein via the venous plexus around the uterus. **d** After embolization, perfusion blood from the left kidney poured into the upper left lumbar vein and the vertebral plexus

**Fig. 4:**
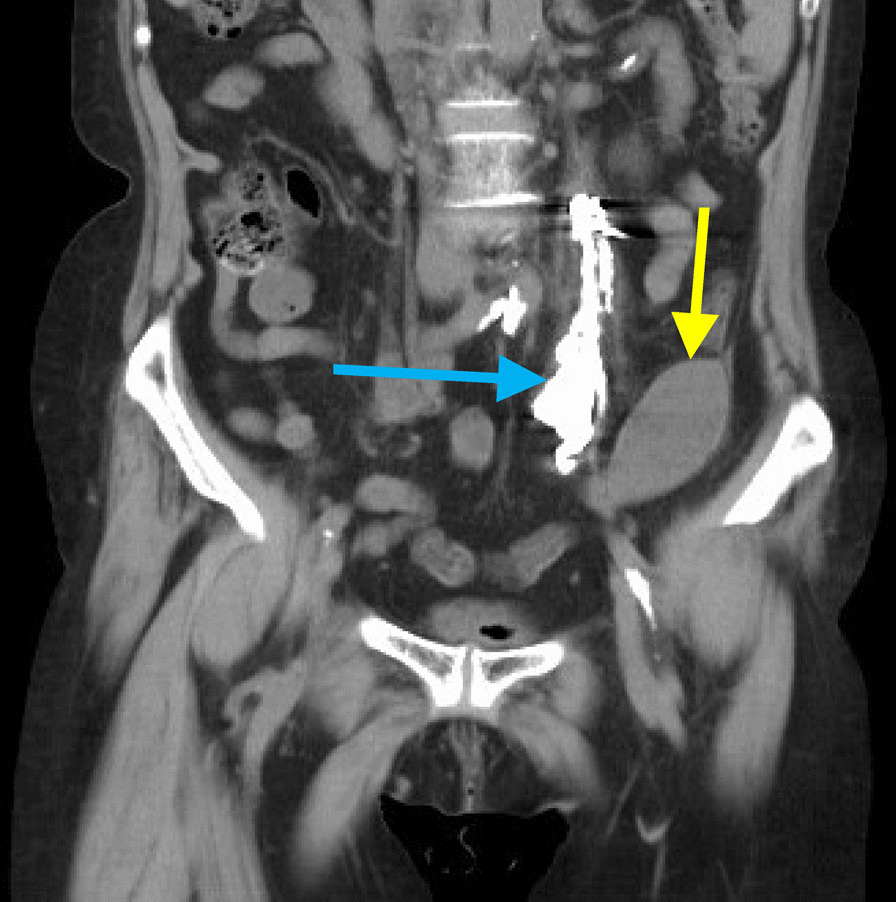
Computed tomographic image on the second day after embolization. The yellow arrow indicates the hematoma, which shrank, and the blue arrow indicates the embolized left ovarian vein

We decided that urgent treatment was needed for her repeated bleeding and performed vascular embolization therapy simultaneously with angiography. However, angiography of the internal iliac artery, including the left uterine artery, and the left external iliac artery, did not show AVM or AVF into the LOV. Angiography of the left renal artery confirmed that the contrast medium discharged from the left kidney did not flow into the inferior vena cava (IVC) but flowed back into the LOV (Fig. 3a). Subsequent venography also confirmed that the blood from the LRV did not flow into the IVC but regurgitated into the LOV and the lumbar vein (Fig. 3b). Left ovarian venography showed retrograde blood flow into a dilated and tortuous aneurysm in the pelvis and into the right internal iliac vein via a vein around the uterus (Fig. 3c). The cause of retroperitoneal bleeding was rupture of the LOV, the distension of which was caused by reflux. We performed embolization of LOV with *N*-butyl-2-cyanoacrylate and placed a coil centrally to prevent recanalization. Postembolization LRV imaging showed blood flow to the upper left lumbar vein and vertebral plexus (Fig. 3d). CT on the second day after the procedure confirmed good embolization of LOV and reduction of the retroperitoneal hematoma to a size of 6.4 cm (Fig. 4). The patient then underwent regular outpatient follow-up, and the retroperitoneal hematoma gradually shrank and disappeared. At her final visit 2 years later, the hematoma had not recurred.

**Fig. 5:**
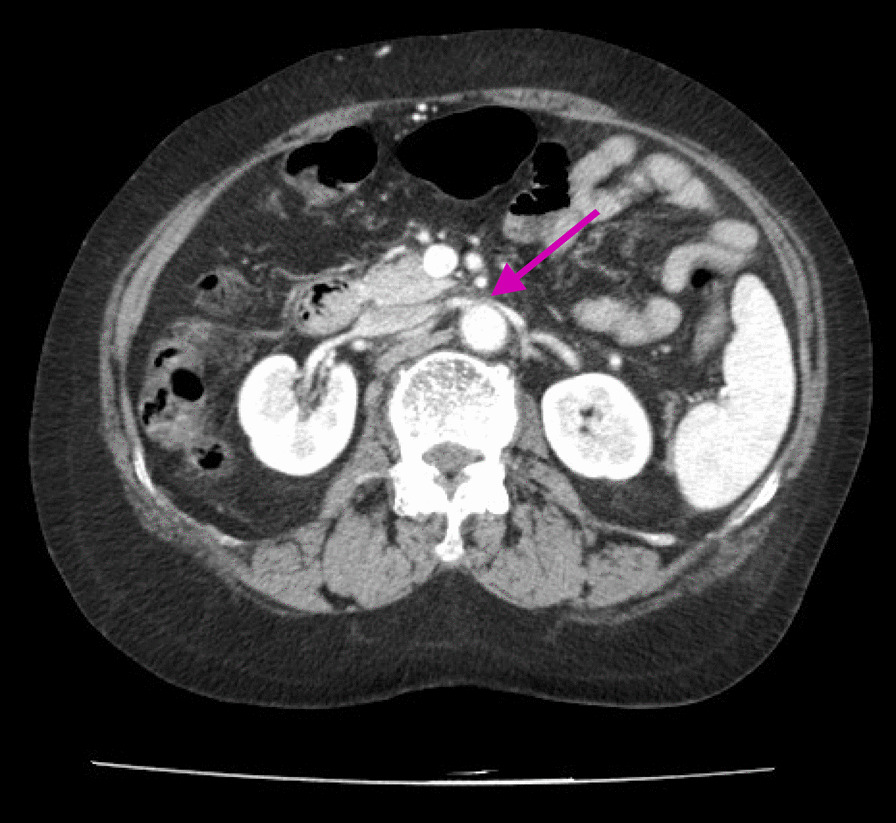
Computed tomographic image shows severe stenosis of the left renal vein. The purple arrow indicates the beak sign

In this case, angiography confirmed the continuity of LRV and IVC but demonstrated no antegrade blood flow to the IVC. Therefore, we attributed LOV regurgitation to extravascular physical compression of LRV. After close examination of the CT images, we determined that LOV rupture was caused by NCS because the LRV was severely stenotic between the abdominal aorta and the SMA (Fig. 5).

## Discussion

This case was an emergency of retroperitoneal hemorrhage with a background of NCS, but it was necessary to rule out various diseases before we could establish the final diagnosis. The referring physician diagnosed her condition as torsion of a left ovarian cyst, and we observed bleeding from what we thought was an AVM or AVF in her pelvis at her first visit. Additional diagnostic imaging and angiography finally demonstrated left ovarian venous regurgitation and rupture. These studies were extensive because of our lack of knowledge of NCS. NCS is not a well-known disease, and it is observed mainly by pediatricians, urologists, vascular surgeons, and radiologists; it is less familiar to gynecologists. In recent years, PCS has begun to attract attention in the field of gynecology as a cause of chronic pelvic pain in women. NCS can be part of the cause of PCS, but alone it is not the same condition [6]. Moreover, this case was further difficult to diagnose because of an extremely rare condition: formation of a spherical para-adnexal mass as a result of spontaneous rupture of an asymptomatic ovarian varicose vein. Prior knowledge of the risk of ovarian vein rupture in association with NCS could have enabled earlier diagnosis and treatment.

Spontaneous rupture of the ovarian vein was, in one case, caused by an abnormal vascular network associated with liver cirrhosis in a patient with a history of ruptured esophageal varices [7], and in a few cases of utero-ovarian vein rupture associated with pregnancy [8, 9]. The only case of reported rupture of the gonadal vein as a result of NCS was spontaneous varicocele rupture in a man [10]. However, we know of no other reports of such rupture caused by NCS. Our patient had no history of trauma, and the cause of the spontaneous rupture was unknown. She had a history of hypertension, but her blood pressure was under good control. She also had a history of hepatitis C but no history of cirrhosis.

NCS diagnosis is confirmed mainly by image evaluation, but no unified diagnostic standard exists [1, 2, 11]. CT and Doppler ultrasonography are the imaging methods most commonly used for diagnosis; the diagnostic criteria are the distance between the SMA and abdominal aorta and the beak sign in axial images, and the angle between SMA and abdominal aorta in sagittal images [1, 4, 11, 12]. Another method of diagnosis is to measure the blood pressure gap between the IVC and LRV, but this method is invasive and uncommon [1, 2, 4, 12]. In this case, we diagnosed NCS because the CT images showed clear LRV stenosis and because venography confirmed regurgitation from the LRV to LOV.

The prevalence of the nutcracker phenomenon ranges from 4.1% to 7.8% among healthy individuals, and of these cases, 8.8% or fewer are symptomatic NCS [2, 11]. Younger age, low BMI, and female sex are risk factors for this illness [1, 11]. Our patient, however, was elderly and had a BMI of 27.4. Also, she did not have any subjective symptoms such as chronic pelvic pain until this episode. CT images showed typical LRV pinching, mild urinary occult blood was confirmed, and left ovarian varicose veins were present, all of which were suggestive of the nutcracker phenomenon.

To treat NCS, the timing and method of intervention must be individualized. For young patients, conservative treatment with monitoring for changes is the first choice, and for mild symptoms, follow-up monitoring for weight gain is the first choice. Although no clear criteria have been established, surgical and endovascular treatment can be used for severe symptoms [1, 2, 4, 13]. In this case, endovascular embolization therapy was performed to treat the emergency caused by rupture. We were concerned about the outflow of LRV after embolization, but the patient did not show any abnormal findings such as hematuria, impaired renal function, or low back pain during later follow-up.

## Conclusion

To our knowledge, spontaneous rupture of the LOV caused by NCS has not been reported in the past; this case is the first report. Because NCS is a disease that has not yet been fully characterized, it should be considered in the differential diagnosis of bleeding emergencies. Knowledge of NCS may be helpful in the appropriate and prompt treatment if an abnormal vascular network in the pelvis is found, especially in bleeding emergencies, such as this case.

## Data Availability

Not applicable.

## Abbreviations

LRV: Left renal vein
SMA: Superior mesenteric artery
NCS: Nutcracker syndrome
PCS: Pelvic congestion syndrome
CT: Computed tomography
BMI: Body mass index
3D-CT: Three-dimensional computed tomography
AVM: Arteriovenous malformation
AVF: Arteriovenous fistula
LOV: Left ovarian vein
IVC: Inferior vena cava
